# Non-invasive quantification of anatomical and functional renal artery vessel wall changes in patients with resistant hypertension undergoing renal denervation using MRI

**DOI:** 10.1186/1532-429X-16-S1-P172

**Published:** 2014-01-16

**Authors:** Adelina Doltra, Arthur Hartmann, Bernhard Schnackenburg, Christopher Schneeweis, Rolf Gebker, Alexander Berger, Philipp Stawowy, Eckart Fleck, Sebastian Kelle

**Affiliations:** 1 Cardiology, German Heart Institute Berlin, Berlin, Germany; 2 Philips Healthcare Systems, Hamburg, Germany

## Background

Renal Denervation (RDN) is a novel therapy for patients with resistant hypertension. Its direct effects on the renal arteries in humans are poorly examined. We sought to assess renal artery distensibility noninvasively using magnetic resonance imaging (MRI) and to study the effects of RDN on anatomical and functional changes of the renal artery vessel wall.

## Methods

19 patients with resistant hypertension undergoing RDN were prospectively included. A 3.0T MRI including contrast-enhanced renal artery angiography was performed before the RDN procedure and at 6-month follow-up. In each patient the proximal part of both renal arteries was imaged for the cross-sectional area measurements using cine spiral MRI. Renal artery sharpness was evaluated with a quantitative analysis tool (Soap-Bubble Tool). In a subgroup of 11 patients, the distensibility (mm Hg(-1) × 10(3)) was determined as (maximum lumen area - minimal lumen area)/(pulse pressure × minimal lumen area). The pulse pressure was calculated as the difference between the systolic and diastolic brachial blood pressure.

## Results

**Figure 1:**
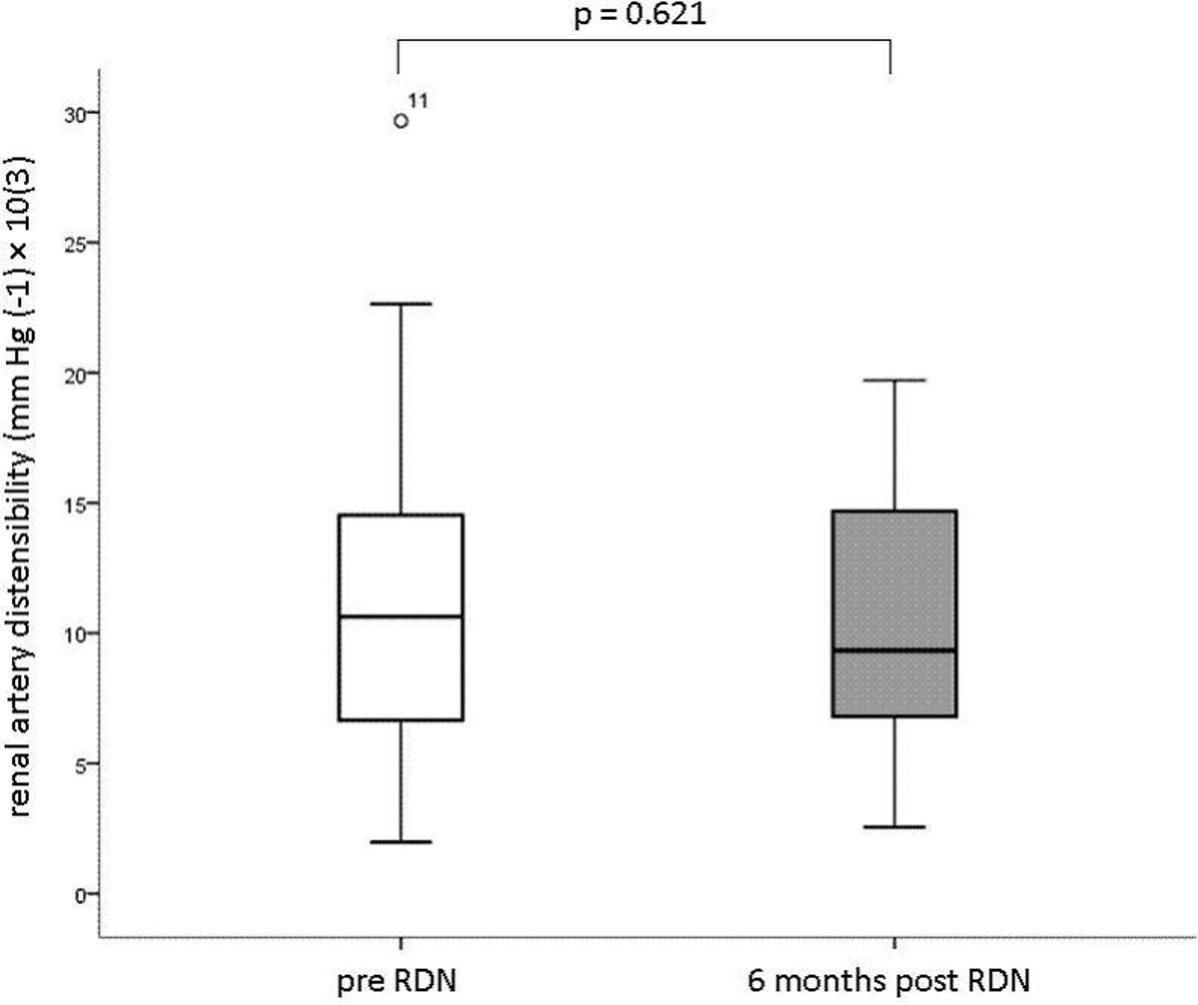
**Box-plots demonstrating renal artery distensibility measured pre and 6-months post renal denervation in patients with resistant hypertention**.

Renal artery sharpness values before and 6 months after RDN did not differ significantly (49.15 ± 7.11 vs 47.43 ± 8.5, respectively, p = 0.255). Similarly, renal artery distensibility was not significantly different before RDN versus at 6-month follow-up (mean ± SD 11.8 ± 7.8 mm Hg(-1) × 10(3) vs 10.6 ± 5.2 mm Hg(-1) × 10(3), respectively, p = 0.621. (FIGURE 1). Minimal and maximal renal artery lumen area increased significantly after RDN compared to baseline, 18.8 ± 5.8 mm2 to 30.9 ± 13.0 mm2 (p = 0.02) respectively 31.7 ± 8.1 mm2 to 51.1 ± 23.2 mm2 (p = 0.01).

## Conclusions

3.0-Tesla MRI, a noninvasive method to assess human renal artery vessel wall sharpness and distensibility, is able to detect no changes in renal artery wall anatomy and function before the RDN procedure and at 6-month follow-up. The effect of enlargement of the renal arteries post RDN suggests a potential effect of RDN on sympathetic tone of the vasculature.

## Funding

None.

